# Secretion of celiac disease autoantibodies after in vitro gliadin challenge is dependent on small-bowel mucosal transglutaminase 2-specific IgA deposits

**DOI:** 10.1186/1471-2172-9-6

**Published:** 2008-02-29

**Authors:** Satumarja M Stenman, Katri Lindfors, Ilma R Korponay-Szabo, Olli Lohi, Päivi Saavalainen, Jukka Partanen, Katri Haimila, Herbert Wieser, Markku Mäki, Katri Kaukinen

**Affiliations:** 1 Medical School, Pediatric Research Center, University of Tampere, Tampere, Finland; 2 Heim Pal Childrens' Hospital, Budapest, Hungary; 3 Department of Medical Genetics, University of Helsinki, Helsinki, Finland; 4 Finnish Red Cross Blood Service, Helsinki, Finland; 5 Deutsche Forschungsanstalt für Lebensmittelchemie, Garching, Germany; 6 Department of Pediatrics, Tampere University Hospital, Tampere, Finland; 7 Department of Gastroenterology and Alimentary Tract Surgery, Tampere University Hospital, Tampere, Finland

## Abstract

**Background:**

In celiac disease gluten, the disease-inducing toxic component in wheat, induces the secretion of autoantibodies which are targeted against transglutaminase 2 (TG2). These autoantibodies are produced in the small-intestinal mucosa, where they can be found deposited extracellularly below the epithelial basement membrane and around mucosal blood vessels. In addition, during gluten consumption these autoantibodies can also be detected in patients' serum but disappear from the circulation on a gluten-free diet. Interestingly, after adoption of a gluten-free diet the serum autoantibodies disappear from the circulation more rapidly than the small-intestinal mucosal autoantibody deposits. The toxicity of gluten and the secretion of the disease-specific autoantibodies have been widely studied in organ culture of small-intestinal biopsy samples, but results hitherto have been contradictory. Since the mucosal autoantibodies disappear slowly after a gluten-free diet, our aim was to establish whether autoantibody secretion to organ culture supernatants in treated celiac disease patient biopsies is related to the duration of the diet and further to the pre-existence of mucosal TG2-specific IgA deposits in the cultured biopsy samples.

**Results:**

In the organ culture system conducted with biopsies derived from treated celiac disease patients, gliadin induced secretion of autoantibodies to culture supernatants, reduced epithelial cell height and increased the density of *lamina proprial *CD25+ cells. However, these changes could be demonstrated only in biopsies from short-term treated celiac disease patients, where the small-intestinal mucosal TG2-specific IgA autoantibody deposits were still present. Furthermore, in these biopsies autoantibody secretion could be stimulated fully only after a 48-hour gliadin challenge.

**Conclusion:**

Our results show that studies focusing on the toxic effects of gliadin in the organ culture system should be carried out with biopsy samples from short-term treated celiac disease patients who are likely still to have mucosal IgA deposits present. In addition to providing an explanation for the discrepancies in previous publications, the present study also enables further validation of the organ culture method.

## Background

Celiac disease is a gluten-induced autoimmune disease of the small intestine characterized by small-bowel mucosal villous atrophy with crypt hyperplasia and a profound inflammation in the mucosa. In addition to causing damage to the mucosa in genetically susceptible individuals, gluten also provokes the production of autoantibodies typically found in the sera of untreated celiac disease patients. These autoantibodies recognize exclusively endomysial antigens now identified as transglutaminase 2 (TG2). The autoantibodies are produced locally in the mucosa [1,2], and besides being detectable in patient sera, they are also deposited extracellularly *in vivo *in the mucosa [3-7]. Furthermore, recent findings suggest that these TG2-targeted mucosal IgA-autoantibody deposits are already present in the early phases of the disease process prior to manifest mucosal lesion [4,5] and before autoantibodies appear in the serum [3-6]. After adoption of a gluten-free diet, serum autoantibodies disappear and the small-bowel mucosa heals usually within one year [8]. At the time the autoantibodies have disappeared from the circulation, there may still be residual autoantibody deposits present in the small-intestinal mucosa which will also in due course disappear on a strict gluten-free diet [4,6].

For over twenty years the human small-intestinal organ culture method has been widely used in detecting the toxic effects of wheat gliadin in celiac disease *in vitro *[1,9-15]. In earlier studies the toxicity of gliadin has commonly been demonstrated by an increased density of *lamina proprial *lymphocytes [11-13] and reduced epithelial cell height (ECH) [14,16] in cultured biopsy samples from untreated and treated celiac disease patients. However, when measuring endomysial autoantibody (EmA) secretion to culture supernatants from biopsies from treated celiac patients, the results have been contradictory. Picarelli and associates [1] showed that in biopsies derived from treated celiac disease patients gliadin induces secretion of EmA to culture supernatants. In contrast, some studies report that the secretion of EmA can only be achieved in half of [14,17] or even no [18] experiments carried out with treated celiac patient biopsies. And even further, it has been suggested that EmA secretion to the organ culture system is totally independent of gliadin challenge [18] and histological damage [14].

Due to these discrepancies among previous studies concerning autoantibody secretion to the organ culture system, our aim was to establish in both short- and long-term treated celiac disease patients whether the antibody secretion to culture supernatants is dependent on the duration of patients' gluten-free diet (GFD). Furthermore, since in celiac disease the small-bowel mucosal extracellular TG2-specific IgA deposits seem to disappear slowly after a gluten-free diet [6], we hypothesized that the autoantibody secretion to supernatants is related to the existence of mucosal TG2-specific IgA deposits in the cultured small-bowel biopsy samples.

## Results

### Celiac autoantibodies

In order to study the baseline serum autoantibody levels of study subjects, EmA and TG2 antibody (TG2-ab) titers were measured. All five untreated celiac disease patients involved had positive EmA (median titer 1:500, range 5–4000) and TG2-ab titers (median titer 58.1, range 4.7–100) in serum. In contrast, all 20 treated celiac disease patients and all six non-celiac controls had normal serum autoantibody levels. Furthermore, all celiac disease patients carried either the HLA DQ2 or the DQ8 haplotype. When the small-bowel biopsies from celiac disease patients were challenged in the organ culture system with peptic-tryptic digest of gliadin (PT-gliadin) for 48 hours, EmA was secreted to the culture supernatants in all five untreated but in only 11 out of the 20 treated celiac cases (Table 1). Moreover, no antibodies were found in supernatants from non-celiac controls (Table 1).

**Table 1:** Endomysial antibodies (EmA) in organ culture supernatants after 48 hours' *in vitro *PT-gliadin challenge in untreated and treated celiac disease patients and non-celiac controls.

	**EmA after 48 h *in vitro *PT-gliadin challenge**	
	
	**Positive**	**Negative**
Untreated celiac disease (n = 5)	100%	
Treated celiac disease (n = 20)	55%	45%
Non-celiac control (n = 6)		100%

**Figure 1:**
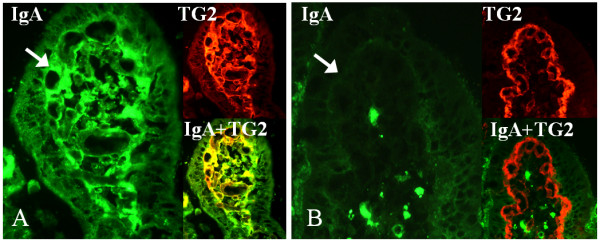
**Small-bowel mucosal transglutaminase 2 (TG2, red)-specific IgA deposits (green) after 24 hours' organ culture**. A) Positive staining (arrow) in the mucosal villous of a short-term treated celiac disease patient (gluten-free diet for three years). B) Negative IgA deposits (arrow) in the small-bowel mucosa of long-term treated celiac disease patient (gluten-free diet for eight years). Co-localization of IgA deposits with TG2 is shown in yellow. Magnification 40×.

When autoantibody secretion to culture supernatants was investigated in relation to the duration of the gluten-free diet, EmA was detected in supernatants after PT-gliadin challenge in 10 out of 12 short-term (GFD 1–3 years) and in 1 out of 8 long-term (GFD 4–20 years) treated patient biopsy samples (Table 2, Table 3). Since the duration of the gluten-free diet was insufficient to explain EmA secretion completely, we tested whether EmA presence in the culture supernatants was dependent on pre-existing small-bowel mucosal IgA-autoantibody deposits. When the treated celiac patients were divided into two groups based on the presence or absence of mucosal IgA autoantibody deposits (Figure 1), it was found that only biopsies derived from patients having positive autoantibody deposits were able to secrete EmA to the culture supernatants (Table 2, Table 3). In some treated celiac cases evincing mucosal IgA deposits, EmA was already secreted to the supernatant spontaneously without PT-gliadin stimulation, but both EmA and TG2 antibody titers increased significantly after 48 hours' culture with PT-gliadin (Table 2). Although the autoantibody titers increased in culture supernatants after PT-gliadin challenge, there was no significant change in the intensity of mucosal IgA deposits in the cultured biopsy samples after the challenge (data not shown).

**Table 2:** Antiendomysial (EmA) and transglutaminase-2 antibody (TG2-ab) secretion to organ culture supernatants after *in vitro *PT-gliadin challenge. Organ culture of treated celiac disease patient biopsy samples (n = 11) who had small-bowel mucosal IgA deposits. Small-intestinal biopsy samples were cultured either with medium only or with PT-gliadin for 24 or 48 hours. EmA titers were graded according to intensity of staining as follows: negative (neg), weak positive (+) and strong positive (++, +++ or ++++).

		**EmA**				**TG2-ab (U)**		
				
**Patient**	**GFD years**	**Medium 24 h**	**PT-gliadin24 h**	**PT-gliadin 48 h**		**Medium 24 h**	**PT-gliadin 24 h**	**PT-gliadin 48 h**
1	1	++	+++	++++		11.0	17.1	37.8
2	1	++	++	+++		7.3	11.8	30.3
3	1	++	++	+++		16.0	6.3	26.6
4	1	+	++	+++		12.4	13.4	9.5
5	1	++	neg	++		17.2	7.4	22.9
6	1	neg	++	++		6.5	13.1	19.9
7	1	neg	neg	+		7.6	5.9	24.5
8	2	neg	+	++		7.8	5.3	14.8
9	3	+	++	++++		21.0	10.3	30.9
10	3	neg	++	++		4.9	7.0	nd
11	4	++	++	nd		24.5	11.9	nd
								
**Median**		**+**	++	+++*	**Median**	**11.0**	**10.3**	**24.5†**

nd = not determined

GFD = gluten-free diet

* P < 0.01; † P = 0.011 compared to medium only 24 hours and PT-gliadin 24 hours.

**Table 3:** Antiendomysial (EmA) and transglutaminase-2 antibody (TG2-ab) secretion to organ culture supernatants after *in vitro *PT-gliadin challenge. Organ culture of treated celiac disease patient biopsy samples (n = 9) who had no small-bowel mucosal IgA deposits. Small-intestinal biopsy samples were cultured either with medium only or with PT-gliadin for 24 or 48 hours. EmA titers were graded according to intensity of staining as follows: negative (neg), weak positive (+) and strong positive (++, +++ or ++++).

		**EmA**				**TG2-ab (U)**		
				
**Patient**	**GFD years**	**Medium 24 h**	**PT-gliadin 24 h**	**PT-gliadin 48 h**		**Medium 24 h**	**PT-gliadin 24 h**	**PT-gliadin 48 h**
12	1	neg	neg	neg		2.0	4.8	13.3
13	2	neg	neg	neg		8.8	6.9	4.4
14	4	neg	neg	neg		2.2	6.2	10.3
15	4	neg	neg	neg		2.5	1.7	2.6
16	6	neg	neg	neg		8.1	7.7	12.4
17	7	neg	neg	neg		2.4	3.7	1.9
18	8	neg	neg	neg		2.5	1.3	1.7
19	14	neg	neg	neg		0.9	2.9	0.7
20	20	neg	neg	neg		11.6	2.5	11.6
								
**Median**		**neg**	**neg**	**neg**	**Median**	**2.5**	**3.7**	4.4*

GFD = gluten-free diet

* P > 0.05 compared to medium only 24 hours and PT-gliadin 24 hours

### Determination of ECH and the number of CD25+ lymphocytes

**Figure 2:**
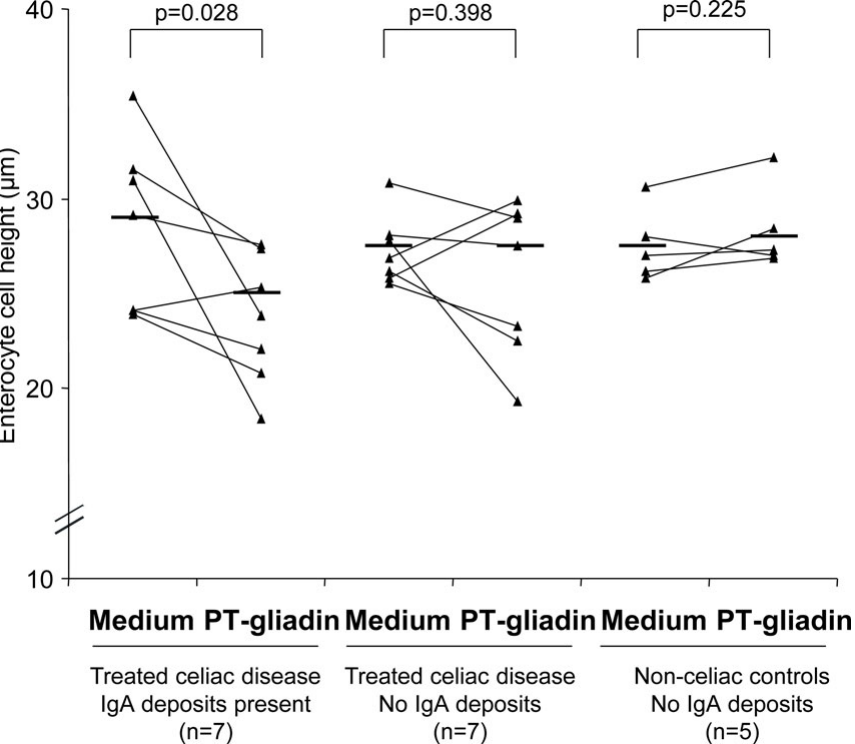
**Enterocyte cell height (ECH) after organ culture**. Biopsy samples of treated celiac disease patients with and without mucosal IgA deposits and non-celiac control patients. Biopsies were cultured with medium only or with PT-gliadin for 24 hours. The median values (horizontal line) and P values are calculated for each group showing a statistically significant decrease in ECH only in those treated celiac disease cases who had small-bowel mucosal IgA deposits in the cultured biopsy samples.

**Figure 3:**
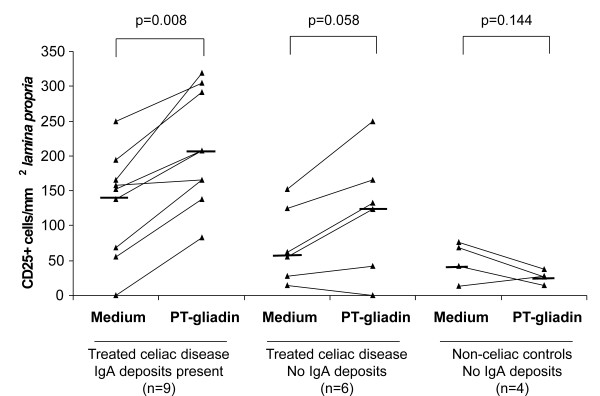
**The total number of mucosal *lamina proprial *CD25-positive T-cells**. Cultured biopsy samples from treated celiac disease patients with and without mucosal IgA deposits and non-celiac control patients. Biopsies were cultured either with medium only or with PT-gliadin for 24 hours. The median values (horizontal line) and P values are calculated for each group showing a statistically significant increase in CD25+ cells only in those treated celiac disease cases who had small-bowel mucosal IgA deposits in the cultured biopsy samples.

In further demonstrating the toxic effects of gliadin, ECH and the number of *lamina proprial *CD25+ cells were calculated in cultured biopsy samples. In summary, it was found that these parameters were also dependent on the presence of mucosal IgA deposits. The ECH decreased and the density of CD25+ cells increased significantly only in the treated celiac disease patient biopsies with pre-existing IgA deposits (Figure 2 and 3).

## Discussion

The results presented here show that gliadin toxicity, as measured by autoantibody secretion, decrease in ECH and increase in the density of *lamina proprial *CD25+ cells, can be demonstrated in the treated celiac disease patient organ culture system only if small-intestinal mucosal TG2-specific IgA autoantibody deposits are still present in the cultured biopsy samples. Patients with persisting mucosal autoantibody deposits, normal small-bowel mucosal villous structure and negative serum autoantibody levels are usually those who have been on a gluten-free diet for a short period of time.

Our results explain the discrepancies reported in previous papers on EmA secretion in biopsies derived from treated celiac disease patients. Vogelsang and colleagues [18] reported that EmA secretion after a 24-hour gliadin challenge was achieved in only 2 out of 18 (11%) biopsy samples derived from celiac disease patients on a strict gluten-free diet with normal mucosal villous morphology. In contrast, biopsies from 60% of patients with dietary lapses and 92% of untreated celiacs with ongoing mucosal inflammation responded to the challenge in respect of EmA secretion. Similar results have been obtained by Biagi and associates [14] in a small-scale study involving only seven treated celiac disease patients. EmA secretion to the culture supernatant could not be stimulated after a 24-hour gliadin administration in any of the long-term (8–30 years) treated celiac patients with normal mucosal architecture. Samples responding to the gliadin insult were derived from patients still evincing mucosal damage despite a gluten-free diet. In the above-mentioned studies the treated patients unable to react to gliadin as measured by EmA secretion were well and long-term treated and may be hypothesized not to have small-bowel IgA autoantibody deposits. This absence of mucosal deposits could thus explain the discrepancy in results. Furthermore, it has been shown that 24 hours of gliadin treatment might not be a sufficient time to induce EmA secretion, as shown by Picarelli and colleagues [19] who demonstrated that a 48-hour culture period in the presence of gliadin is needed to achieve EmA secretion in all treated celiac disease patient samples, this also being in agreement with the results presented in the current study (Table 2).

It remains to be elucidated how a gliadin challenge leads to EmA secretion in the organ culture system. Basically there are two different possibilities. Firstly, EmA could be actively secreted by plasma cells in the biopsy specimen [1]. The inability of long-term treated patient biopsy samples to secrete autoantibodies to the supernatant could be explained by the overall absence of plasma cells and helper T cells as well as the failure of memory B cells to become activated within the timeframe of the organ culture [17,18]. Secondly, the secretion of EmA to the culture supernatant might simply be due to detachment of the autoantibodies from the tissue deposits and their release into the supernatant. However, we observed no decrease in the intensity of the mucosal autoantibody deposits after the organ culture. We did nevertheless see changes in ECH and the density of CD25+ cells, which speaks in favor of an active gliadin-induced process, this supporting the first proposition.

## Conclusion

Our results indicate that when studying the toxic effects of gliadin in an organ culture setting, biopsy samples from short-term treated celiac disease patients who are likely still to have mucosal TG2-specific IgA deposits should be used. Altogether, the current study provides a platform for further validation of the organ culture method.

## Methods

### Patients

Small-bowel mucosal biopsies were taken from 20 treated patients suffering from celiac disease (median age 54 years, range 23–73 years, females 80%), five untreated celiac patients (median age 48 years, range 43–71 years, females 100%) and six non-celiac control subjects suffering from dyspepsia (median age 53 years, range 24–70 years, females 50%). In all celiac patients the diagnosis was initially based on the European Society of Pediatric Gastroenterology and Nutrition criteria [20], meaning that they all had small-bowel mucosal villous atrophy with crypt hyperplasia in the duodenum while consuming gluten. All treated celiac patients involved in the current study had been on a strict gluten-free diet for at least one year (median duration of GFD three years, range 1–20 years), and all, as well as the non-celiac controls, showed normal small-bowel mucosal architecture. In contrast, all the untreated patients evinced subtotal villous atrophy with crypt hyperplasia in the small-bowel mucosa. The study protocol was accepted by the Ethical Committee of Tampere University Hospital and written informed consent was obtained from all patients and controls.

### Small-bowel mucosal biopsies and organ culture system

Altogether seven small-bowel mucosal biopsy samples were obtained from each patient during upper gastrointestinal endoscopy. Two samples were immediately snap-frozen in liquid nitrogen with optimal cutting temperature compound (OCT, Tissue-Tek, Sakura Finetek Europe, Holland) and stored at -20°C until used. Further, two biopsies were immediately fixed in paraffin for investigation of the baseline small-bowel mucosal morphology. The remaining three biopsies were cultured for 24 or 48 hours at 37°C, either in the presence or absence of a peptic-tryptic digest of gliadin (1 mg/ml) prepared by a standard protocol described elsewhere [15,21].

The organ culture method was implemented as originally described by Browning and Trier [9]. Briefly, the biopsy samples were cultured in RPMI-1640 medium (Invitrogen-Gibco, Paisley, Scotland, UK) containing 15 % heat-inactivated fetal bovine serum (Invitrogen-Gibco), 100 μg/ml streptomycin (Invitrogen-Gibco), 100 U/ml penicillin (Invitrogen-Gibco), 4 mM L-glutamine (Invitrogen-Gibco), 50 μg/ml insulin (Sigma-Aldrich Co, St. Louis, Missouri, USA) and 10 mM HEPES buffer (Invitrogen-Gibco). Duodenal specimens were placed villi upwards on a sterile stainless-steel grid positioned over the medium in a central well of the organ culture dish (Falcon, Becton Dickinson and Co, USA). After 24 or 48 hours' incubation, culture supernatants were collected and stored at -70°C until analysed. Free fluid was removed from the samples, whereafter they were snap-frozen with OCT and stored at -20°C until processed for stainings.

### Celiac autoantibodies

EmA was detected in patients' serum and undiluted organ culture supernatants using an indirect immunofluorescence assay where human umbilical cord was used as antigen [8]. A serum dilution of 1:≥5 was considered positive. Antibody titers for organ culture supernatants were graded according to the intensity of the staining as follows: negative (neg), weak positive (+) and strong positive (++, +++ or ++++). Samples were analyzed blindly without knowledge of the patients' clinical background.

TG2-antibodies were measured by enzyme-linked immunosorbent assay (ELISA, Celikey^®^, Phadia, Freiburg, Germany), according to manufacturer's instructions, both in serum samples (diluted 1:100) and in undiluted culture supernatants. In serum samples a unit value (U) ≥ 5U was considered positive. Since there is no cut-off value for TG2-antibody in culture supernatants, the crude antibody values are given only for comparison to EmA.

### Small-bowel mucosal TG2-specific IgA deposits

The small-bowel mucosal TG2-targeted IgA deposits were investigated before and after 24 hours of organ culture. In earlier studies it has been shown that these mucosal IgA deposits are specifically targeted against TG2 in the small-bowel mucosa [3,6]. In the studies in question, autoantibody specificity for TG2 was demonstrated by the fact that IgA eluted from duodenal mucosa bound intensively to purified TG2 in ELISA and Western blot [3]. Similarly, a human recombinant TG2 was capable of depositing celiac-specific IgA in small-bowel sections from celiac disease patients [6]. In addition, after removal of TG2 from the sections by a specific acid, both TG2 and IgA deposits disappeared from the mucosa [6].

To study the mucosal IgA deposits the 5-μm-thick unfixed cryostat sections were stained using a two-color immunofluorescence method as previously described [3]. The monoclonal primary antibody against TG2 (Clone CUB 7402, NeoMarkers, Fremont, USA, 1:200) was used followed by the rhodamine-conjugated antimouse immunoglobulin antibody (Dako, A/S, Glostrup, Denmark 1:120) and the fluorescein isothiocyanate-conjugated rabbit antibody against human IgA (Dako, 1:40). In untreated celiac disease a clear extracellular subepithelial IgA deposition can be found below the basement membrane along the villous and crypt epithelium and around mucosal vessels; this is in contrast to non-celiac normal small-bowel samples, where IgA is detected only inside plasma and epithelial cells [4,6,22].

### Determination of ECH and the number of CD25+ lymphocytes

ECH was measured under a light microscope (Olympus BX60, 40× magnification) after 24 hours' organ culture with or without PT-gliadin challenge using the analySIS 3.0 program, (Soft Imaging System GmbH, Munster, Germany). Altogether 30 enterocytes from three different villi of each specimen were analyzed and a mean ECH value was calculated for each biopsy sample [14].

CD25-positive T cells were detected in the *lamina propria *of small-bowel mucosa from biopsy samples cultured for 24 hours with or without PT-gliadin. The 5-μm-thick cryostat sections were fixed in acetone and incubated with goat normal serum (Vector Laboratories Inc., Burlingame, USA), whereafter they were incubated with mouse monoclonal antibody, human anti-CD25 (Dako, 1:25) for one hour and alexa-conjugated goat anti-mouse IgG (Invitrogen, 1:1000) for 30 minutes. Washes with PBS were performed between each antibody. The density of small-bowel mucosal CD25-positive T-cells in the *lamina propria *was calculated and presented as number of cells in a total area of one mm^2 ^of *lamina propria *[12,13].

### HLA-typing

Celiac disease is strongly associated with the HLA gene region, since over 95% of celiac disease patients have either HLA DQ2 or HLA DQ8 haplotype molecules [23,24]. HLA-DQ typing was performed in each patient using DELFIA^® ^Celiac Disease Hybridization Assay (PerkinElmer Life and Analytic Sciences, Wallac Oy, Turku, Finland).

### Statistical analysis

Statistical analysis was performed using 2-tailed Wilcoxon Signed Ranks Test or Mann-Whitney U Test, as appropriate. P values lower than 0.05 were considered statistically significant.

## Authors' contributions

SS designed the study, carried out the experiments, collected data and drafted the manuscript

KL designed the study, supervised the work, participated in the writing of the manuscript and provided funding

IKS designed the study, analyzed the data and revised the manuscript

OL designed the study and revised the manuscript

PS performed the HLA-typing of patients and revised the manuscript

JP performed the HLA-typing of patients and revised the manuscript

KH performed the HLA-typing of patients and revised the manuscript

HW prepared the PT-gliadin used in the experiments and revised the manuscript

MM originated the idea for the research, provided funding and revised the manuscript

KK supervised the work, studied the patients and participated in the writing of the manuscript

All authors have read and approved the final manuscript.

## References

[B1] Picarelli A, Maiuri L, Frate A, Greco M, Auricchio S, Londei M (1996). Production of antiendomysial antibodies after in-vitro gliadin challenge of small intestine biopsy samples from patients with coeliac disease. Lancet.

[B2] Marzari R, Sblattero D, Florian F, Tongiorgi E, Not T, Tommasini A, Ventura A, Bradbury A (2001). Molecular dissection of the tissue transglutaminase autoantibody response in celiac disease. J Immunol.

[B3] Korponay-Szabo IR, Halttunen T, Szalai Z, Laurila K, Kiraly R, Kovacs JB, Fesus L, Maki M (2004). In vivo targeting of intestinal and extraintestinal transglutaminase 2 by coeliac autoantibodies. Gut.

[B4] Kaukinen K, Peraaho M, Collin P, Partanen J, Woolley N, Kaartinen T, Nuutinen T, Halttunen T, Maki M, Korponay-Szabo I (2005). Small-bowel mucosal transglutaminase 2-specific IgA deposits in coeliac disease without villous atrophy: A prospective and randomized clinical study. Scand J Gastroenterol.

[B5] Salmi TT, Collin P, Jarvinen O, Haimila K, Partanen J, Laurila K, Korponay-Szabo IR, Huhtala H, Reunala T, Maki M, Kaukinen K (2006). Immunoglobulin A autoantibodies against transglutaminase 2 in the small intestinal mucosa predict forthcoming coeliac disease. Aliment Pharmacol Ther.

[B6] Salmi TT, Collin P, Korponay-Szabo IR, Laurila K, Partanen J, Huhtala H, Kiraly R, Lorand L, Reunala T, Maki M, Kaukinen K (2006). Endomysial antibody-negative coeliac disease: clinical characteristics and intestinal autoantibody deposits. Gut.

[B7] Hadjivassiliou M, Maki M, Sanders DS, Williamson CA, Grunewald RA, Woodroofe NM, Korponay-Szabo IR (2006). Autoantibody targeting of brain and intestinal transglutaminase in gluten ataxia. Neurology.

[B8] Sulkanen S, Halttunen T, Laurila K, Kolho KL, Korponay-Szabo IR, Sarnesto A, Savilahti E, Collin P, Maki M (1998). Tissue transglutaminase autoantibody enzyme-linked immunosorbent assay in detecting celiac disease. Gastroenterology.

[B9] Browning TH, Trier JS (1969). Organ culture of mucosal biopsies of human small intestine. J Clin Invest.

[B10] Jos J, Lenoir G, Ritis GD, Rey J (1975). In vitro pathogenetic studies of coeliac disease. Effects of protein digests on coeliac intestinal biopsy specimens maintained in culture for 48 hours. Scand J Gastroenterol.

[B11] Maiuri L, Picarelli A, Boirivant M, Coletta S, Mazzilli MC, De Vincenzi M, Londei M, Auricchio S (1996). Definition of the initial immunologic modifications upon in vitro gliadin challenge in the small intestine of celiac patients. Gastroenterology.

[B12] Maiuri L, Ciacci C, Ricciardelli I, Vacca L, Raia V, Auricchio S, Picard J, Osman M, Quaratino S, Londei M (2003). Association between innate response to gliadin and activation of pathogenic T cells in coeliac disease. Lancet.

[B13] Salvati VM, Mazzarella G, Gianfrani C, Levings MK, Stefanile R, De Giulio B, Iaquinto G, Giardullo N, Auricchio S, Roncarolo MG, Troncone R (2005). Recombinant human interleukin 10 suppresses gliadin dependent T cell activation in ex vivo cultured coeliac intestinal mucosa. Gut.

[B14] Biagi F, Parnell ND, Ellis HJ, Ciclitira PJ (2000). Endomysial antibody production is not related to histological damage after in vitro gluten challenge. Eur J Gastroenterol Hepatol.

[B15] Kilmartin C, Lynch S, Abuzakouk M, Wieser H, Feighery C (2003). Avenin fails to induce a Th1 response in coeliac tissue following in vitro culture. Gut.

[B16] Shidrawi RG, Day P, Przemioslo R, Ellis HJ, Nelufer JM, Ciclitira PJ (1995). In vitro toxicity of gluten peptides in coeliac disease assessed by organ culture. Scand J Gastroenterol.

[B17] Carroccio A, Iacono G, D'Amico D, Cavataio F, Teresi S, Caruso C, Di PL, Colombo A, D'Arpa F, Florena A, Notarbartolo A, Montalto G (2002). Production of anti-endomysial antibodies in cultured duodenal mucosa: usefulness in coeliac disease diagnosis. Scand J Gastroenterol.

[B18] Vogelsang H, Schwarzenhofer M, Granditsch G, Oberhuber G (1999). In vitro production of endomysial antibodies in cultured duodenal mucosa from patients with celiac disease. Am J Gastroenterol.

[B19] Picarelli A, Sabbatella L, Di Tola M, Vetrano S, Maffia C, Picchi C, Mastracchio A, Paoluzi P, Anania MC (2001). Forty-eight hours of biopsy culture improve the sensitivity of the in vitro gliadin challenge in the diagnosis of celiac disease. Clin Chem.

[B20] (1990). Revised criteria for diagnosis of coeliac disease. Report of Working Group of European Society of Paediatric Gastroenterology and Nutrition. Arch Dis Child.

[B21] Bolte G, Osman A, Mothes T, Stern M (1996). Peptic-tryptic digests of gliadin: contaminating trypsin but not pepsin interferes with gastrointestinal protein binding characteristics. Clin Chim Acta.

[B22] Korponay-Szabo IR, Laurila K, Szondy Z, Halttunen T, Szalai Z, Dahlbom I, Rantala I, Kovacs JB, Fesus L, Maki M (2003). Missing endomysial and reticulin binding of coeliac antibodies in transglutaminase 2 knockout tissues. Gut.

[B23] Polvi A, Arranz E, Fernandez-Arquero M, Collin P, Maki M, Sanz A, Calvo C, Maluenda C, Westman P, de la Concha EG, Partanen J (1998). HLA-DQ2-negative celiac disease in Finland and Spain. Hum Immunol.

[B24] Sollid LM, Markussen G, Ek J, Gjerde H, Vartdal F, Thorsby E (1989). Evidence for a primary association of celiac disease to a particular HLA-DQ alpha/beta heterodimer. J Exp Med.

